# Agreement of Bioelectrical Impedance Analysis Prediction Equations with Dual-Energy X-Ray Absorptiometry for Tracking Resistance Training-Induced Changes in Fat-Free Mass in Older Women

**DOI:** 10.3390/sports14100421

**Published:** 2026-10-01

**Authors:** Alex S. Ribeiro, André N. S. Cahebo, Amilton T. Canguali, Felipe Lisboa, Witalo Kassiano, Paolo M. Cunha, Matheus A. Nascimento, Diogo V. Martinho, Analiza M. Silva, Luís B. Sardinha, Manuel João Coelho-e-Silva, Edilson S. Cyrino

**Affiliations:** 1 University of Coimbra, CIPER, FCDEF, 3040-248 Coimbra, Portugal; amiltonteixeiracanguali@gmail.com (A.T.C.); dvmartinho92@hotmail.com (D.V.M.); mjcesilva@hotmail.com (M.J.C.-e.-S.); 2 Higher Technical School of Sports Sciences (ESCID), Luanda, Angola; andrecahebo@gmail.com; 3 Metabolism, Nutrition, and Exercise Laboratory, Physical Education and Sport Center, State University of Londrina, Londrina 86057-970, PR, Brazil; felipe.lisboa7@uel.br (F.L.); acc.witalo@gmail.com (W.K.); pcunha88@hotmail.com (P.M.C.); edilsoncyrino@gmail.com (E.S.C.); 4 University Pitágoras Unopar Anhanguera, Londrina 86041-120, Brazil; 5 Department of Physical Education, Paraná State University, Unespar, Paranavaí Campus, Paranavaí 87703-000, PR, Brazil; matheus.nascimento@unespar.edu.br; 6 Exercise and Health Laboratory, CIPER, Faculdade Motricidade Humana, Universidade de Lisboa, 1499-002 Lisbon, Portugal; analiza.monica@gmail.com (A.M.S.); lbsardinha55@gmail.com (L.B.S.)

**Keywords:** body composition, aging, strength training, measurement error, agreement analysis

## Abstract

This study examined the agreement between commonly used bioelectrical impedance analysis (BIA) prediction equations and dual-energy X-ray absorptiometry (DXA) for tracking resistance training-induced changes in fat-free mass (FFM) in older women. One hundred and five older women (68.4 ± 5.8 years) completed a 24-week supervised resistance training program. FFM was assessed before and after training using DXA and estimated using eight BIA prediction equations. DXA-derived FFM increased significantly after training (Δ = 0.9 ± 2.3 kg). All BIA equations also detected significant increases in FFM, with mean changes similar to those observed with DXA after multiplicity adjustment (*p* > 0.05). However, this similarity at the group level did not translate into agreement at the individual level. Correlations between BIA equation-derived and DXA-derived changes were weak to moderate (r = 0.26–0.42), and despite generally small mean biases (−0.25 to 0.59 kg), the 95% limits of agreement were wide across all equations. Moreover, significant negative proportional bias was observed for all equations, indicating progressively greater underestimation relative to DXA as the magnitude of FFM change increased. Collectively, these findings demonstrate a clear distinction between the ability of BIA prediction equations to reproduce mean group-level changes and their ability to track individual changes in FFM. Thus, although BIA prediction equations may be useful for describing average group-level adaptations to resistance training in older women, they should not be considered interchangeable with DXA for monitoring individual changes in FFM.

## 1. Introduction

Aging is accompanied by marked alterations in body composition, particularly a progressive decline in fat-free mass (FFM), which is closely associated with reductions in muscle strength, functional capacity, metabolic health, independence, and longevity in older adults [1,2,3]. On the other hand, resistance training effectively counters age-related muscle loss and promotes increases in FFM, even in advanced age [4,5]. Accurate monitoring of FFM changes is essential for clinical decision-making and for evaluating exercise interventions in older adults. Small changes in FFM may translate into meaningful differences in physical function, metabolic health, and independence, particularly in aging populations in whom baseline muscle mass is already reduced [6,7]. Imprecise assessment methods may lead to misinterpretation of intervention effectiveness, inappropriate adjustments in training prescription, or incorrect assumptions regarding treatment response. Therefore, beyond cross-sectional validity, body composition methods must demonstrate sufficient agreement and sensitivity to detect longitudinal changes in FFM at the individual level, especially when used to guide clinical or exercise-related decisions.

Dual-energy X-ray absorptiometry (DXA) is often regarded as a reference method for assessing FFM and detecting longitudinal changes in FFM [8,9]. Despite its recognized validity, DXA is limited by cost, accessibility, and the need for specialized facilities, which restrict its routine use in many applied and clinical settings. In contrast, bioelectrical impedance analysis (BIA) offers a practical, low-cost, and noninvasive alternative widely used in both research and clinical settings. Even though BIA devices are practical, their FFM estimates rely heavily on prediction equations. Variability in predictive equations may be influenced by factors including the specific BIA equation used, device type, and population characteristics such as age and training status [10]. Moreover, while some predictive equations can correlate well with DXA at single time points, their accuracy for tracking changes in body composition following an intervention program can be inconsistent [11,12,13,14,15]. Although numerous predictive equations have been developed for older adults, most were derived from samples of untrained individuals [10].

Previous studies have shown that BIA equations vary in their ability to track FFM changes following resistance training in older women, with some demonstrating acceptable agreement with DXA and others showing limited concordance [11,14]. For example, in a previous investigation, Nascimento et al. [14] examined the agreement between BIA-derived equations and DXA for tracking changes in FFM following resistance training in older women. The observed results indicated that, whereas some equations can detect training-induced changes in FFM at the group level, others exhibit limited accuracy, suggesting substantial heterogeneity among equations [14]. However, the intervention period in that study was relatively short, which may have constrained the magnitude of measurable FFM changes and limited the ability to fully assess the equations’ longitudinal sensitivity. Changes in lean mass in older adults are time-dependent, and total training time is an important moderator of the hypertrophy response [16]. Thus, longer resistance training interventions are more likely to elicit measurable and consistent hypertrophic adaptations, underscoring the importance of intervention duration when evaluating the ability of body composition methods to detect accurate changes.

Therefore, the primary aim of the present study was to examine the agreement between individual changes in FFM estimated by eight BIA-based prediction equations and those assessed by DXA following 24 weeks of resistance training in older women. As secondary aims, we examined the association between individual change scores and each equation’s ability to detect the mean group-level increase in FFM induced by resistance training. We hypothesized that BIA equations would reproduce mean group-level changes in FFM but would show limited agreement with DXA for individual change scores.

## 2. Materials and Methods

### 2.1. Experimental Design

This analysis used data from the Active Aging Longitudinal Study, an ongoing research program investigating the effects of supervised resistance training on health-related outcomes in older women. The present study pooled data from four cohorts recruited and assessed between 2022 and 2024, comprising 105 participants who completed 24 weeks of resistance training. The four cohorts contributed 32, 26, 27, and 20 participants, respectively. Across cohorts, the training intervention, equipment, assessors, testing procedures, and laboratory conditions were standardized. Measurements were performed at baseline (weeks 1–3) and post-intervention (weeks 28–30), following the 24-week resistance training period (weeks 4–27). At both time points, assessments were conducted in the morning, at the same time of day for each participant, following a 12-h overnight fast. Participants were instructed to abstain from alcohol and caffeine for at least 48 h before testing. BIA was performed first, followed by DXA on a separate day, with at least 24 h between assessments. Post-intervention testing was initiated at least 72 h after the last resistance training session. None of the participants included in these four cohorts participated in the previous study by Nascimento et al. [14]; therefore, there was no participant overlap between the two studies.

### 2.2. Participants

A total of 119 women were enrolled and started the resistance training program. The sample size was determined by the availability of eligible participants with complete data from the longitudinal study. During the 24-week intervention, 14 participants discontinued for personal reasons unrelated to the study, resulting in 105 participants who completed the intervention. All completers met the required attendance (≥85%) and had complete data for the outcomes analyzed; therefore, no additional participants were excluded for insufficient attendance or missing measurements. The final analysis included 105 participants. Participants completed health history and physical activity questionnaires and met specific inclusion criteria: (a) female; (b) over 60 years old; (c) physically independent; (d) free from cardiac or orthopedic issues; (e) not undergoing hormonal replacement or thyroid therapy; and (f) not engaged in more than one exercise session per week in the previous 6 months. Only those who completed at least 85% of resistance training sessions and provided complete data were included. After being informed about the procedures, benefits, and risks, participants signed consent forms. All procedures adhered to the Declaration of Helsinki and received approval from the University Ethics Committee.

### 2.3. Anthropometry

Body mass was recorded to the nearest 0.1 kg using a calibrated electronic scale (Balmak, model Class III, Labstore, Curitiba, PR, Brazil), while height was measured to the nearest 0.1 cm with a stadiometer attached to the scale. Participants wore light workout clothing and no shoes during both measurements. Body mass index was computed by dividing body mass in kilograms by the square of height in meters.

### 2.4. Dual-Energy X-Ray Absorptiometry

A Lunar Prodigy Advance device (GE Lunar, Madison, WI, USA), model NRL 419900, was used to determine fat-free and fat mass via a total-body scan. FFM was calculated as the sum of whole-body lean soft tissue and bone mineral content obtained from the DXA analysis using enCORE software, version 18.0 (GE HealthCare, Madison, WI, USA). Participants removed all metal objects before the scan and were positioned supine along the center of the scanning table, with their feet taped together at the toes to minimize movement and their hands maintained in a pronated position within the scan area. Participants were instructed to remain completely still throughout the procedure. An experienced technician performed device calibration and scan analysis according to the manufacturer’s instructions. The software-generated lines separating the limbs from the trunk and head were inspected and, when necessary, manually adjusted by the technician according to predefined anatomical landmarks, following the manufacturer’s recommendations. The same technician performed all analyses throughout the intervention and was blinded to the assessment time point. All measurements were performed in the morning after an overnight fast, with room temperature maintained between 19 and 23 °C.

### 2.5. Bioimpedance

The BIA measurements were performed using a single-frequency device operating at 50 kHz (BIA/Vitality Analyzer™, The Nutritional Solutions Corporation Ltd.; Harrisville, MI, USA). Resistance (R) and reactance (Xc) were estimated, and phase angle was then calculated as arctangent (Xc/R) × 180°/π [17]. All bioelectrical impedance measurements took place in the morning. Before assessments, the device was calibrated according to the manufacturer’s procedures. Participants removed all metal objects. Measurements were conducted on an electrically isolated table, with subjects lying supine along the table’s centerline, hips abducted at 45° from the midline, and hands pronated. After skin cleaning with alcohol, two electrodes were placed on the right hand and two on the right foot. Participants were instructed to urinate about 30 min before testing, follow a 12-h overnight fast, refrain from strenuous exercise for at least 24 h, avoid alcohol and caffeine for at least 48 h, and not use diuretics for the week prior. The same professional conducted all measurements during both pre- and post-intervention sessions. No additional device firmware or software-version information was available in the study records.

The equations proposed by Deurenberg et al. [18], Dey et al. [19], Gray et al. [20], Kyle et al. [21], Masset et al. [22], Roubenoff et al. [23], Sun et al. [24], and Williams et al. [25] were used to estimate fat-free mass (FFM). All equations used in this experiment are displayed in Table 1. We selected the equations based on their applicability to older adults, methodological diversity in reference methods and predictor variables, and the availability of all variables required to calculate each equation in its original form at both pre- and post-training assessments. All selected equations included older individuals in their development or validation samples, although some were derived specifically from older populations and others from broader adult populations.

### 2.6. Resistance Training Program

All participants followed the same supervised resistance training routine for 24 weeks, held in the mornings on Mondays, Wednesdays, and Fridays at the university facilities. The protocol followed the National Strength and Conditioning Association’s guidelines to enhance muscle endurance and strength in older adults [5]. This full-body program targeted major muscle groups through eight exercises: chest press, horizontal leg press, seated row, leg extension, preacher curl (using free weights), seated leg curl, triceps pushdown, and seated calf raise. The range of motion was standardized for each exercise. For the chest press, participants lowered the handles until the elbows reached approximately 90° of flexion and then extended the elbows without locking them. Participants performed the horizontal leg press from approximately 90° of knee flexion to near-full knee extension, without locking the knees. During the seated row, participants started with the elbows extended and pulled the handles toward the trunk until the elbows moved slightly behind the torso. The leg extension was performed from approximately 90° of knee flexion to near-full knee extension, whereas the seated leg curl was performed from near-full knee extension to approximately 90° of knee flexion. The preacher curl was performed from near-full elbow extension to maximal comfortable elbow flexion while maintaining the upper arms supported on the bench. The triceps pushdown was performed from approximately 90° of elbow flexion to near-full elbow extension. Finally, the seated calf raise was performed from maximal comfortable ankle dorsiflexion to maximal plantar flexion. Participants were instructed to perform each repetition through the prescribed range of motion while maintaining proper technique, and exercise execution was supervised throughout all training sessions.

During the first 12 weeks, participants completed three sets of 10–15 repetitions per exercise, shifting to 8–12 repetitions in the last 12 weeks. Initial training loads were established by trial and error to identify a load that allowed participants to perform repetitions within the prescribed repetition range with appropriate technique. All sets were performed to voluntary muscular failure. Rest intervals between sets were 1–2 min, and rest between exercises lasted 2–3 min. Participants were instructed to inhale during eccentric movements and exhale during concentric movements, maintaining a 1:2 speed ratio (concentric to eccentric). Exercise loads were tailored to each participant’s ability and progress in exercise capacity during the study. The goal was to ensure participants exercised with maximum feasible resistance while maintaining proper technique. When participants could complete 15 repetitions (first 12 weeks) or 12 repetitions (last 12 weeks) across all sets in two consecutive sessions for a given exercise, the weight was increased by 2–5% for upper limb exercises and 5–10% for lower limb exercises in the subsequent session [26].

### 2.7. Statistical Analysis

Distributional assumptions were examined using the Shapiro–Wilk test and inspection of standardized within-participant change scores (post-training minus pre-training). Body mass and phase-angle change scores did not deviate significantly from normality (*p* > 0.05), whereas significant departures were observed for the remaining longitudinal variables (*p* < 0.05). A small number of extreme standardized change scores (>3 SD from the mean) were identified in several variables; therefore, nonparametric Wilcoxon signed-rank sensitivity analyses were also performed for the pre- to post-training FFM comparisons and yielded the same inferential conclusions as the parametric analyses. Pre- and post-training FFM values were analyzed using a two-way repeated-measures analysis of variance (ANOVA), with time (pre- and post-training) and method (DXA and the eight BIA equations) as within-subject factors. When the assumption of sphericity was violated, the Greenhouse–Geisser correction was applied. Bonferroni-adjusted pairwise comparisons were used to examine differences between time points and methods. Absolute changes in FFM were compared across methods using a repeated-measures one-way ANOVA. Agreement between equation-derived and DXA-derived changes in FFM was evaluated using Bland–Altman analysis [27], including mean bias and 95% limits of agreement (LoA). Approximate 95% confidence intervals for the lower and upper LoA were calculated using the standard error of each LoA (SD of the differences × √(3/*n*)). Proportional bias was assessed by linear regression of the between-method differences (equation minus DXA change) on the mean of the two change estimates; a slope significantly different from zero indicated proportional bias. Pearson’s correlation coefficient was used to examine associations between equation-derived and DXA-derived FFM at baseline, after training, and over time. The agreement analyses comparing each BIA equation with DXA were considered the primary analyses. Correlation analyses involving bioelectrical impedance parameters, SWC-based summaries, and other complementary analyses were considered exploratory; because these analyses were intended to characterize patterns rather than provide confirmatory hypothesis tests, their *p* values were not adjusted for multiplicity and should be interpreted cautiously. Correlation strengths were categorized as very weak (0.00–0.20), weak (0.21–0.40), moderate (0.41–0.60), strong (0.61–0.80), and very strong (≥0.81). Effect size for paired differences was calculated as Cohen’s d [28] (mean difference divided by the standard deviation of the paired differences) and classified as trivial (<0.20), small (0.20–0.49), moderate (0.50–0.79), or large (≥0.80). The smallest worthwhile change (SWC) was defined as 0.2 times the standard deviation of change in FFM for each method. Additionally, the proportion of participants exceeding the method-specific SWC was calculated for DXA and each BIA equation as an exploratory descriptive index of the observed changes. Statistical significance was set at *p* < 0.05 for the primary analyses. All analyses were performed using IBM SPSS Statistics (version 28.0; IBM Corp., Armonk, NY, USA).

## 3. Results

Participant characteristics and changes in anthropometric, body composition, and bioelectrical impedance parameters following the 24-week resistance training intervention are presented in Table 2. Body mass, height, and BMI did not change significantly over the intervention period (*p* > 0.05), whereas fat mass and percentage body fat decreased significantly (*p* < 0.05). Regarding bioelectrical impedance parameters, R and R/height decreased significantly, while Xc, Xc/height, and phase angle increased significantly from pre- to post-training (*p* < 0.001).

Table 3 presents FFM values derived from DXA and estimated using the eight BIA prediction equations before and after the resistance training intervention, together with their respective changes over time. DXA-derived FFM increased significantly following resistance training (*p* < 0.05). At baseline, FFM estimates from all BIA equations except Kyle et al. differed significantly from DXA-derived FFM after Bonferroni adjustment (*p* < 0.05). At post-training, FFM estimates from all BIA equations differed significantly from DXA-derived values (*p* < 0.05). Nevertheless, all BIA equations detected significant pre- to post-training increases in FFM (*p* < 0.05), and the mean ΔFFM estimated by the equations did not differ significantly from that derived from DXA after Bonferroni adjustment (*p* > 0.05). Thus, despite differences in absolute FFM estimates, the BIA equations reproduced the direction and magnitude of the mean group-level change observed with DXA.

Table 4 presents the correlations between FFM estimated using the BIA prediction equations and DXA-derived FFM at pre-training and post-training, as well as the correlations between changes over time. At baseline, correlations between BIA equation-derived and DXA-derived FFM were very strong for most equations and strong for Gray et al. (r = 0.838–0.885, *p* < 0.001). Post-training correlations were very strong for all equations (r = 0.872–0.919, *p* < 0.001). In contrast, correlations between BIA equation-derived and DXA-derived ΔFFM were weak to moderate but statistically significant for all equations (r = 0.261–0.416, *p* ≤ 0.007). Thus, the strong cross-sectional associations observed for absolute FFM values were not maintained when individual changes in FFM were examined.

Table 5 summarizes the agreement between ΔFFM estimated using the BIA prediction equations and DXA-derived ΔFFM. Mean biases were generally small, ranging from −0.25 to 0.59 kg across equations. However, the 95% limits of agreement were wide for all equations, with lower limits ranging from −4.69 to −4.25 kg and upper limits from 3.91 to 5.44 kg; the corresponding confidence intervals are provided in Table 5. Bland–Altman plots for each equation are presented in Supplementary Figure S1. Significant negative proportional bias was observed for all equations (all *p* ≤ 0.002), indicating that the equation-minus-DXA difference became progressively more negative as the magnitude of FFM change increased. Therefore, the relatively small mean biases at the group level were accompanied by substantial variability and systematic magnitude-dependent disagreement at the individual level.

The smallest worthwhile change (SWC) ranged from 0.25 to 0.34 kg across the BIA equations. Effect sizes for the differences between BIA equation-derived and DXA-derived ΔFFM were trivial for all equations except Kyle et al., for which a small effect was observed. Using DXA-derived ΔFFM, 71.4% of participants exceeded the SWC. When equation-specific SWC values were applied, the corresponding proportions were 84.8% for Deurenberg et al., 90.5% for Dey et al., 87.6% for Gray et al., 87.6% for Kyle et al., 89.5% for Masset et al., 92.4% for Roubenoff et al., 84.8% for Sun et al., and 86.7% for Williams et al.

Table 6 presents the correlations between raw and height-adjusted bioelectrical impedance parameters and DXA-derived FFM at pre-training and post-training, as well as between their respective changes over the intervention period. At baseline, DXA-derived FFM was negatively correlated with R, Xc, R/height, and Xc/height. Similar negative associations were observed at post-training. Changes in DXA-derived FFM were also significantly and negatively correlated with changes in R, Xc, R/height, and Xc/height.

## 4. Discussion

This study examined the agreement of eight BIA equations for tracking resistance-training-induced changes in FFM in older women, using DXA as the reference method. The main finding was that although all BIA-based equations detected significant group-level increases in FFM after 24 weeks of resistance training in older women, substantial variability remained in their agreement and individual-level accuracy relative to DXA-derived changes. Given the large number of BIA predictive equations available for different populations, we selected eight equations a priori based on their applicability to the variables available in our dataset and their relevance to older women [10]. Importantly, these equations differ in their derivation populations; some were developed specifically for older adults, whereas others were generalized adult equations derived from broader age ranges that included older individuals. None, however, were developed specifically for resistance-trained older women. This heterogeneity in derivation populations, together with differences in reference methods and predictor variables, may influence their ability to track longitudinal changes in FFM.

Differences in longitudinal performance across BIA prediction equations may reflect heterogeneity in their derivation populations, reference methods, predictor variables, and mathematical structure. Among the equations examined, Sun et al.’s equation [24] showed the smallest mean bias for ΔFFM (−0.04 kg). Although differences in the predictors included in each equation, such as R, Xc, and the H^2^/R index, could potentially contribute to differences in their estimates, the present study was not designed to determine which equation characteristics account for their longitudinal performance. Therefore, mechanistic explanations for the lower mean bias observed with Sun et al.’s equation would be speculative. More importantly, a small mean bias should not be interpreted as evidence of good agreement at the individual level. Despite its near-zero mean bias, Sun et al.’s equation showed wide 95% limits of agreement (−4.38 to 4.30 kg), indicating substantial variability in the discrepancy between equation-derived and DXA-derived changes across individuals. Thus, the performance of Sun et al.’s equation illustrates the central distinction observed in the present study: an equation may closely reproduce the mean group-level change in FFM while still showing limited agreement with DXA for tracking individual changes.

The Xc reflects the capacitive properties of cell membranes and is commonly interpreted as an indicator of cellular integrity [29]. The Xc has been shown to be a useful predictor in cross-sectional equations, particularly in aging populations, because it declines with age, which explains its incorporation into predictive models such as those proposed by Kyle et al. [21] and Roubenoff et al. [23]. In the present analysis, however, the equations including raw Xc did not show a consistent pattern of mean bias: Kyle et al.’s equation showed the largest positive mean bias (0.59 kg), whereas Roubenoff et al.’s equation showed a comparatively small mean bias (0.17 kg). Accordingly, the present findings do not support attributing longitudinal agreement to the inclusion or exclusion of Xc alone, because these equations also differ in coefficients, derivation samples, reference methods, and other predictors. Resistance training increased Xc at the group level, consistent with previous findings [30]; however, changes in Xc were negatively correlated with changes in DXA-derived FFM. These findings are not contradictory, as the former represents the mean response over time, whereas the latter describes the relationship between individual changes. Thus, although Xc increased on average, participants with larger FFM increases tended to show smaller Xc increases. Other experiments also observed a negative association between FFM and Xc. For example, Marini et al. [31] reported significant negative associations between Xc and FFM, with a correlation indicating that individuals with higher FFM tend to have lower Xc values in BIA analyses (r = −0.33). Similar patterns have been observed by Brunani et al. [32], in which populations with higher body mass and FFM were associated with reductions in Xc values. The present study did not directly assess plausible physiological explanations for the observed changes. Therefore, whether including Xc affects prediction equations’ ability to track longitudinal changes in FFM remains uncertain and warrants further investigation.

The equations proposed by Dey et al. [19], Gray et al. [20], Masset et al. [22], Roubenoff et al. [23], Sun et al. [24], and Williams et al. [25] exhibited comparatively small mean biases. Those by Dey et al. [19], Sun et al. [24], and Williams et al. [25] were derived using four-compartment (4C) models, which reduce some assumptions inherent in simpler body composition models; however, equations derived using densitometry or DXA also showed small mean biases. Therefore, the present findings do not allow differences in longitudinal performance to be attributed specifically to the reference method used to derive each equation. Other characteristics, including derivation samples, predictor variables, and equation coefficients, may also contribute to the observed differences. An additional finding from the Bland–Altman analysis was significant proportional bias for all equations. Although mean biases were generally small, the negative proportional bias indicates that the discrepancy between equation-derived and DXA-derived changes was not constant across the range of FFM changes. Specifically, as the magnitude of FFM change increased, the equations tended to provide progressively lower ΔFFM estimates relative to DXA. Thus, a small mean bias should not be interpreted as evidence of consistent agreement across individuals, particularly among those exhibiting larger changes in FFM.

Another important factor relates to the population used to derive predictive equations. Applying predictive equations to individuals whose characteristics differ from those of the population in which the equation was originally developed increases the risk of systematic bias. Therefore, interpreting the performance of BIA equations outside their source population needs some caution. In the present study, all equations included older individuals during their development. However, some were derived from mixed populations comprising both young and older adults, whereas others were developed exclusively in older individuals. Notably, all predictive equations were originally derived from untrained populations. Consequently, applying these equations to individuals with different physiological characteristics, such as those undergoing resistance training, may increase the likelihood of systematic estimation errors. Resistance training induces several physiological adaptations that directly affect the electrical properties measured by BIA, particularly R and Xc, by altering the conductive properties of FFM components [30]. Because many predictive equations assume relatively stable electrical properties derived from untrained individuals, training-induced changes in body water distribution and tissue electrical conductivity may violate these assumptions, thereby reducing the longitudinal accuracy of some equations when applied to resistance-trained individuals.

Some studies have focused on verifying changes in the FFM using BIA equations in older populations [11,14]. Nascimento et al. [14] studied 43 older women over 12 weeks of resistance training, comparing FFM changes measured by DXA with single-frequency BIA device output and six BIA prediction equations. Several equations systematically over- or under-estimated absolute FFM vs. DXA, both before and after training. After training, only two equations detected statistically significant FFM gains of a magnitude similar to DXA, while the others showed no significant change from pre- to post-training. Importantly, the intervention period was relatively short (12 weeks), which may have constrained the magnitude of detectable changes in FFM and limited the assessment of the equations’ longitudinal sensitivity. In addition, only a limited number of predictive equations were examined, restricting the generalizability of the findings given the wide range of BIA equations currently available for older populations. Moon et al. [11] investigated healthy older adults undergoing resistance training and reported that both DXA and single-frequency BIA showed good agreement with the four-compartment (4C) model for estimating group-level changes in FFM. However, the ability to track individual changes was limited, particularly when variations were smaller than 5 kg. In a subgroup of older women undergoing 24 weeks of resistance training, DXA detected FFM changes very similar to those observed with the 4C model (4C = 0.83 ± 1.3 kg; DXA = 0.84 ± 0.84 kg). In contrast, the BIA predictive equations exhibited substantial variability in estimating FFM changes and generally underestimated values compared with DXA and the 4C model, with differences ranging from 0.01 to 0.85 kg.

Our experiment observed an average change in FFM of 0.9 kg, which aligns with a meta-analysis showing an increase of 0.9 kg (95% CI 0.6, 1.3) in FFM induced by RT in older individuals [33]. Nascimento et al. [14] detected a mean FFM gain of ~0.65 ± 0.82 kg after 12 weeks of resistance training. The greater increase observed in the current experiment than in Nascimento et al. [14] may be related to the intervention duration. Total training duration is a key moderator of hypertrophic adaptations in older adults, as longer resistance training programs are associated with greater and more consistent increases in FFM [16]. Accordingly, extending the intervention duration provides a more robust framework for determining whether BIA equations can reliably track resistance training-induced changes in FFM when compared with DXA.

The method-specific SWCs and the proportions of participants exceeding these thresholds were considered exploratory descriptive indices and should not be interpreted as evidence of individual-level sensitivity or responder classification. Exceeding the SWC indicates only that the observed change surpassed a method-specific threshold and does not demonstrate that the magnitude or direction of change agrees with DXA. Moreover, because laboratory-specific measurement error was unavailable, these distribution-based SWC thresholds cannot establish whether an observed individual change exceeded the technical error of either measurement method. Therefore, the relatively high proportions of participants exceeding the SWC can coexist with the limited individual-level agreement indicated by the Bland–Altman analyses.

Importantly, these limitations in longitudinal monitoring should not be interpreted as questioning the clinical utility of BIA. BIA is widely used as a practical, non-invasive, and accessible point-of-care method for cross-sectional body composition assessment across different clinical populations. Indeed, numerous predictive equations have been developed for specific populations, including older adults and individuals with chronic diseases, highlighting the broad clinical applicability of this method [10]. Recent studies further illustrate this utility; for example, Greco et al. [34] used BIA-derived estimates of FFM and skeletal muscle mass to assess body composition and muscular fitness in adults with type 1 diabetes. However, cross-sectional profiling and longitudinal monitoring have different requirements. While BIA may provide clinically useful body composition estimates, its ability to detect small individual changes depends on measurement error relative to the expected change. Thus, our findings do not challenge the clinical value of BIA but suggest caution when using predictive equations to monitor small individual changes in FFM following resistance training.

Some limitations of the present study should be acknowledged. First, the analyses were restricted to participants who completed the intervention and had complete data, which may have introduced selection bias. In addition, participants were previously untrained older women; therefore, the findings may not be directly generalizable to resistance-trained or more physically conditioned individuals, who may exhibit different magnitudes of FFM change and bioelectrical characteristics. Importantly, laboratory-specific repeated-measure precision estimates for DXA and BIA were not consistently available across all cohorts. This limitation is particularly relevant when evaluating longitudinal changes at the individual level because the magnitude of an observed change should be interpreted relative to the measurement error of the method. Consequently, we were unable to determine whether individual changes in FFM exceeded the expected technical measurement error of DXA or BIA or to calculate laboratory-specific thresholds such as typical error or least significant change. Thus, some of the variability observed in individual change scores and in the Bland–Altman limits of agreement may reflect measurement variability in addition to differences between methods. Accordingly, the present findings should be interpreted as evidence of limited agreement between BIA prediction equations and DXA for quantifying individual changes rather than as definitive evidence that a given equation failed to detect a true physiological change. Furthermore, the prediction equations evaluated were originally developed using different populations, devices, predictor variables, and reference methods, which may have influenced their longitudinal performance in the present sample. The exploratory analyses involved multiple comparisons without adjustment for multiplicity and should therefore be interpreted cautiously. Likewise, the method-specific SWCs were distribution-based exploratory thresholds and should not be interpreted as indicators that an individual change exceeded measurement error or represented a true physiological response. Finally, the relatively modest magnitude and range of training-induced changes in FFM may have limited the evaluation of equation performance across larger individual changes, and the absence of an independent replication sample warrants confirmation of these findings in other cohorts. Several longitudinal change scores departed from normality and a small number of extreme standardized changes were identified. However, Wilcoxon signed-rank sensitivity analyses yielded the same conclusions for pre- to post-training FFM changes, suggesting that the main group-level findings were not dependent on the normality assumption.

Despite the aforementioned limitations, the present study has several important strengths. To our knowledge, this is one of the few investigations to evaluate the longitudinal agreement of a broad set of BIA equations during a relatively long resistance-training intervention in older women. The 24-week training duration represents a substantial advance over previous short-term studies and provides a more robust physiological stimulus for evaluating the ability of equations to track meaningful changes in fat-free mass. In addition, including multiple equations developed or validated in older populations enables a comprehensive, comparative assessment of equation performance, highlighting equation-specific differences in bias, agreement, and patterns of change. The combined use of correlation analysis, Bland–Altman agreement, and complementary descriptive indices of variability provides a multidimensional evaluation of the longitudinal performance of BIA equations. Together, these strengths enhance the study’s methodological rigor and contribute novel, clinically meaningful evidence to the literature on body composition assessment in aging populations.

## 5. Conclusions

Our results indicate that BIA prediction equations reproduced the mean group-level increase in FFM observed with DXA following 24 weeks of resistance training in older women. However, this similarity at the group level did not translate into agreement at the individual level, as indicated by weak-to-moderate associations between change scores, significant negative proportional bias, and wide limits of agreement. Because laboratory-specific repeated-measure precision estimates were unavailable, the contribution of measurement error to individual changes could not be quantified. Therefore, BIA prediction equations may be useful for describing average group-level adaptations but should not be considered interchangeable with DXA for quantifying individual changes in FFM.

## Figures and Tables

**Table 1:** Bioelectrical impedance prediction equations.

Author	Equation	Reference Method
Deurenberg et al. (1990) [18]	FFM = 0.340 × H^2^/R + 0.288 × BM − 0.116 × age + 3.5 × sex + 0.097 × H − 3.79	Densitometry
Dey et al. (2003) [19]	FFM = 11.78 + 0.499 × H^2^/R + 0.134 × BM + 3.449 × sex	4-Compartment
Gray et al. (1989) [20]	FFM = 20.387 + 0.00151 × H^2^ − 0.0344 × R + 0.14 × BM − 0.158 × age	Densitometry
Kyle et al. (2001) [21]	FFM = −4.104 + 0.518 × H^2^/R + 0.231 × BM + 0.130 × Xc + 4.229 × sex	DXA
Masset et al. (2025) [22]	FFM = −8.395 + 0.340 × H^2^/R − 5.760 × sex + 0.222 × BM − 0.041 × age + 0.138 × H + 0.700 × PhA	DXA
Roubenoff et al. (1997) [23]	FFM = 7.7435 + 0.4542 × H^2^/R + 0.1190 × BM + 0.0455 × Xc	DXA
Sun et al. (2003) [24]	FFM = −9.53 + 0.69 × H^2^/R + 0.17 × BM + 0.02 × R	4-Compartment
Williams et al. (1995) [25]	FFM = 0.37 × H^2^/R + 0.16 × BM + 11.94	4-Compartment

Note. FFM = fat-free mass in kg, H = height in cm, R = resistance from bioimpedance in ohms, Xc = reactance from bioimpedance in ohms, PhA = phase angle in degrees, BM = body mass in kg. Age is presented in years. Sex coding is reported only for equations that include sex as a predictor: Masset et al., men = 0 and women = 1; Deurenberg et al., Dey et al., and Kyle et al., men = 1 and women = 0.

**Table 2:** General characteristics of the sample (*n* = 105).

Variables	Pre-Training	Post-Training
Age (years)	68.4 ± 5.8	NA
Body mass (kg)	69.2 ± 12.9	69.4 ± 12.8
Height (cm)	155.8 ± 5.9	155.7 ± 5.8
BMI (kg/m^2^)	28.4 ± 4.3	28.5 ± 4.3
Fat mass (kg)	28.9 ± 8.2	28.3 ± 8.1 *
Percentage fat mass (%)	40.8 ± 5.3	40.1 ± 5.1 *
Resistance (Ω)	577.7 ± 61.9	551.1 ± 57.1 **
Resistance/Height (Ω/m)	371.3 ± 43.2	354.5 ± 40.2 **
Reactance (Ω)	50.4 ± 8.3	53.6 ± 7.8 **
Reactance/Height (Ω/m)	32.4 ± 5.7	34.5 ± 5.4 **
Phase angle (°)	5.0 ± 0.7	5.6 ± 0.6 **

Note. BMI = body mass index. NA = not applicable. * *p* < 0.05 vs. pre-training and ** *p* < 0.001 vs. pre-training.

**Table 3:** Fat-free mass (kg) at pre- and post-training according to equations.

Source	Pre-Training	Post-Training	Δ Pre vs. Post (95% CI)
DXA	40.2 ± 6.0	41.1 ± 5.6 *	0.9 ± 2.3 (0.4; 1.3)
Deurenberg	37.8 ± 6.0 #	38.5 ± 5.8 *#	0.6 ± 1.3 (0.4; 0.9)
Dey	42.3 ± 4.4 #	43.3 ± 4.4 *#	1.0 ± 1.7 (0.7; 1.3)
Gray	36.1 ± 5.4 #	36.9 ± 5.2 *#	0.8 ± 1.6 (0.5; 1.1)
Kyle	40.5 ± 5.2	42.0 ± 5.2 *#	1.5 ± 1.7 (1.1; 1.8)
Masset	37.9 ± 5.2 #	39.0 ± 5.1 *#	1.1 ± 1.4 (0.8; 1.3)
Roubenoff	37.6 ± 3.8 #	38.7 ± 3.8 *#	1.1 ± 1.5 (0.8; 1.3)
Sun	43.2 ± 5.1 #	44.1 ± 5.1 *#	0.8 ± 1.6 (0.5; 1.2)
Williams	38.8 ± 4.0 #	39.5 ± 3.9 *#	0.8 ± 1.3 (0.5; 1.0)

Note. * *p* < 0.05 vs. pre-training and # *p* < 0.05 vs. DXA.

**Table 4:** Pearson correlations between fat-free mass estimated by bioelectrical impedance equations and DXA at pre- and post-training and for changes (Δ).

Source	Pre-Training		Post-Training		Δ	
	r	*p*	r	*p*	r	*p*
Deurenberg	0.875	<0.001	0.913	<0.001	0.416	<0.001
Dey	0.875	<0.001	0.872	<0.001	0.381	<0.001
Gray	0.838	<0.001	0.873	<0.001	0.317	<0.001
Kyle	0.874	<0.001	0.919	<0.001	0.261	0.007
Masset	0.876	<0.001	0.919	<0.001	0.309	0.001
Roubenoff	0.873	<0.001	0.887	<0.001	0.328	<0.001
Sun	0.880	<0.001	0.898	<0.001	0.395	<0.001
Williams	0.885	<0.001	0.897	<0.001	0.402	<0.001

**Table 5:** Agreement and descriptive indices of change between bioelectrical impedance equations and DXA-derived changes in fat-free mass.

Source	Bias (kg) (95% CI)	LoA 95% (kg) (95% CI)	SWC	ES	Proportional-Bias Slope (*p*)
Deurenberg	−0.25 ± 2.12 (−0.66 to 0.16)	−4.41 (−5.12 to −3.70); 3.91 (3.20 to 4.62)	0.27	−0.12	−0.71 (<0.001)
Dey	0.12 ± 2.26 (−0.32 to 0.55)	−4.32 (−5.08 to −3.56); 4.55 (3.79 to 5.31)	0.33	0.05	−0.46 (<0.001)
Gray	−0.11 ± 2.33 (−0.56 to 0.34)	−4.69 (−5.47 to −3.90); 4.46 (3.68 to 5.25)	0.31	−0.05	−0.56 (<0.001)
Kyle	0.59 ± 2.47 (0.11 to 1.07)	−4.25 (−5.08 to −3.42); 5.44 (4.61 to 6.27)	0.34	0.24	−0.47 (0.002)
Masset	0.18 ± 2.30 (−0.26 to 0.63)	−4.32 (−5.09 to −3.55); 4.68 (3.91 to 5.45)	0.29	0.08	−0.69 (<0.001)
Roubenoff	0.17 ± 2.28 (−0.27 to 0.61)	−4.29 (−5.05 to −3.53); 4.63 (3.87 to 5.40)	0.29	0.08	−0.66 (<0.001)
Sun	−0.04 ± 2.21 (−0.47 to 0.39)	−4.38 (−5.12 to −3.64); 4.30 (3.56 to 5.04)	0.32	−0.02	−0.52 (<0.001)
Williams	−0.13 ± 2.13 (−0.54 to 0.28)	−4.30 (−5.02 to −3.59); 4.04 (3.33 to 4.76)	0.25	−0.06	−0.79 (<0.001)

Note. Bias was calculated as equation minus DXA. Positive values indicate overestimation, whereas negative values indicate underestimation relative to DXA. SWC = smallest worthwhile change. ES = effect size. Effect size was calculated as the mean difference between equation-derived and DXA-derived changes divided by the standard deviation of the paired differences. Effect size classification: trivial < 0.20, small = 0.20–0.49, moderate = 0.50–0.79, large ≥ 0.80.

**Table 6:** Pearson correlations between bioelectrical impedance parameters and fat-free mass at baseline, post-training, and changes after the intervention (*n* = 105).

Variable	Pre-Training	Post-Training	Δ
Resistance	−0.588 **	−0.475 **	−0.299 **
Reactance	−0.415 **	−0.250 *	−0.388 **
Resistance/Height	−0.724 **	−0.654 **	−0.281 **
Reactance/Height	−0.514 **	−0.393 **	−0.378 **

Note. Values expressed as Pearson correlation coefficient (r). * *p* < 0.05 and ** *p* < 0.01.

## Data Availability

The data presented in this study are available from the corresponding author upon reasonable request. The data are not publicly available due to privacy and ethical restrictions.

## Supplementary Materials

The following supporting information can be downloaded at: https://www.mdpi.com/article/10.3390/sports14100421/s1. Figure S1. Bland–Altman plots comparing changes in fat-free mass (ΔFFM) estimated by bioelectrical impedance analysis equations with changes assessed by dual-energy X-ray absorptiometry (DXA) following 24 weeks of resistance training. Panels represent the equations of (A) Deurenberg et al., (B) Dey et al., (C) Gray et al., (D) Kyle et al., (E) Masset et al., (F) Roubenoff et al., (G) Sun et al., and (H) Williams et al. The solid horizontal line represents the mean bias (equation-derived ΔFFM − DXA-derived ΔFFM), and the dotted lines represent the 95% limits of agreement. The thin solid regression line represents the relationship between the differences and the corresponding mean values.
