# Supplementary material for: Agreement of Bioelectrical Impedance Analysis Prediction Equations with Dual-Energy X-Ray Absorptiometry for Tracking Resistance Training-Induced Changes in Fat-Free Mass in Older Women
Source: Sports (Basel). 2026 Oct 1;14(10):421. doi: 10.3390/sports14100421 (PMC13709518; doi:10.3390/sports14100421)
Supplement: Supplementary file 1 [file sports-14-00421-s001.zip › sports-4448356-supplementary.pdf]

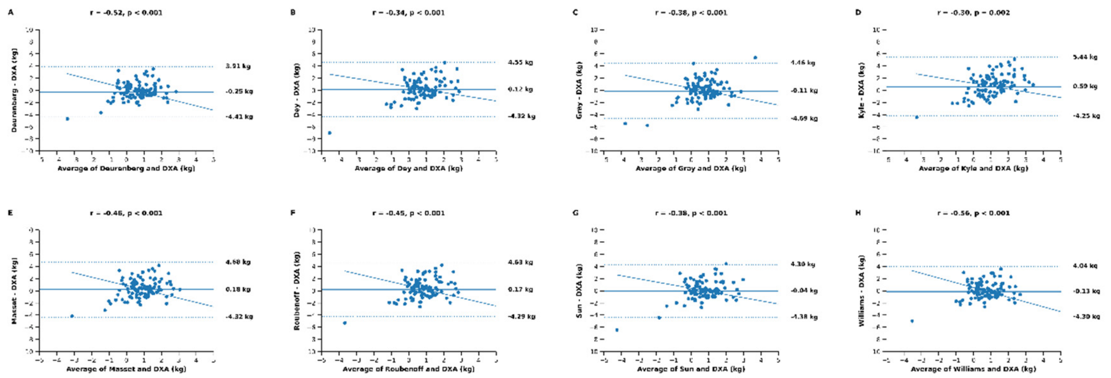

Figure S1. Bland–Altman plots comparing changes in fat-free mass (ΔFFM) estimated by bioelectrical impedance analysis equations with changes assessed by dual-energy X-ray absorptiometry (DXA) following 24 weeks of resistance training. Panels represent the equations of (A) Deurenberg et al., (B) Dey et al., (C) Gray et al., (D) Kyle et al., (E) Masset et al., (F) Roubenoff et al., (G) Sun et al., and (H) Williams et al. The solid horizontal line represents the mean bias (equation-derived ΔFFM − DXA-derived ΔFFM), and the dotted lines represent the 95% limits of agreement. The thin solid regression line represents the relationship between the differences and the corresponding mean values.
